# Patient-physician discussions about costs: definitions and impact on cost conversation incidence estimates

**DOI:** 10.1186/s12913-016-1353-2

**Published:** 2016-03-31

**Authors:** Wynn G. Hunter, Ashley Hesson, J. Kelly Davis, Christine Kirby, Lillie D. Williamson, Jamison A. Barnett, Peter A. Ubel

**Affiliations:** Duke University, School of Medicine, 4906 Glendarion Drive, Durham, NC 27713 USA; Michigan State University, College of Human Medicine, East Lansing, MI USA; Duke University, Fuqua School of Business, Durham, NC USA; University of Illinois, Department of Communication, Champaign, IL USA; Verilogue Incorporated, Horsham, PA USA; Duke University, Sanford School of Public Policy, Durham, NC USA

**Keywords:** Cost of illness, Health expenditures, Out-of-pocket costs, Patient-physician communication, Medical decision-making, Patient-centered care

## Abstract

**Background:**

Nearly one in three Americans are financially burdened by their medical expenses. To mitigate financial distress, experts recommend routine physician-patient cost conversations. However, the content and incidence of these conversations are unclear, and rigorous definitions are lacking. We sought to develop a novel set of cost conversation definitions, and determine the impact of definitional variation on cost conversation incidence in three clinical settings.

**Methods:**

Retrospective, mixed-methods analysis of transcribed dialogue from 1,755 outpatient encounters for routine clinical management of breast cancer, rheumatoid arthritis, and depression, occurring between 2010–2014. We developed cost conversation definitions using summative content analysis. Transcripts were evaluated independently by at least two members of our multi-disciplinary team to determine cost conversation incidence using each definition. Incidence estimates were compared using Pearson’s Chi-Square Tests.

**Results:**

Three cost conversation definitions emerged from our analysis: (a) Out-of-Pocket (OoP) Cost -- discussion of the patient’s OoP costs for a healthcare service; (b) Cost/Coverage -- discussion of the patient’s OoP costs or insurance coverage; (c) Cost of Illness-- discussion of financial costs or insurance coverage related to health or healthcare. These definitions were hierarchical; OoP Cost was a subset of Cost/Coverage, which was a subset of Cost of Illness. In each clinical setting, we observed significant variation in the incidence of cost conversations when using different definitions; breast oncology: 16, 22, 24 % of clinic visits contained cost conversation (OOP Cost, Cost/Coverage, Cost of Illness, respectively; *P <* 0.001); depression: 30, 38, 43 %, (*P <* 0.001); and rheumatoid arthritis, 26, 33, 35 %, (*P <* 0.001).

**Conclusions:**

The estimated incidence of physician-patient cost conversation varied significantly depending on the definition used. Our findings and proposed definitions may assist in retrospective interpretation and prospective design of investigations on this topic.

**Electronic supplementary material:**

The online version of this article (doi:10.1186/s12913-016-1353-2) contains supplementary material, which is available to authorized users.

## Background

Despite recent reductions in national healthcare expenditures, nearly 1 in 3 Americans are financially burdened by their medical expenses [1, 2]. Beyond just financial consequences, high out-of-pocket medical costs have been associated with lower quality of life, delayed or forgone care, and increased risk of adverse health outcomes [2–7]. Our group and others have recommended that physicians and patients engage in routine discussions about healthcare costs during clinic visits, in order to help patients avoid unnecessary out-of-pocket expenditures [8–12]. Theoretically, such discussions could allow physicians and patients to weigh medical and financial trade-offs of treatment or diagnostic options, making choices that optimize patients’ health outcomes while minimizing avoidable financial distress.

Despite these calls to action, there remains a paucity of data informing current understanding of physician-patient cost communication [12]. Little is known about the content of cost conversations and their incidence is unclear, with widely varying estimates in the published literature. In fact, incidence estimates range from 14–15 % of patients ever discussing cost with their physicians [13, 14], to 44–65 % discussing it with their physicians in the previous year alone [15, 16]. This heterogeneity is likely due to a combination of factors, including but not limited to: clinical context, socioeconomic status of the patient population, and mode of inquiry (e.g. physician survey, patient survey, analysis of recorded clinical encounters).

Additionally, differences in ‘cost conversation’ definition or criteria may also contribute to the observed variation in cost conversation incidence estimates. In this paper, we first provide a critical review of cost conversation definitions used in prior studies, highlighting the need for more rigorous definitions. We then describe our development of novel cost conversation definitions through mixed-methods analysis of 1,755 outpatient encounters for routine clinical management of three different diseases (breast cancer, rheumatoid arthritis, and depression). Finally, we report the cost conversation incidence in our sample using each definition, to evaluate the impact of definitional variation on cost conversation incidence estimates. In doing so, we provide clarifying insights for retrospective interpretation of existing literature and future investigation on this important aspect of patient experience [1, 11, 17].

## Methods

### Sample description

Visit transcripts were sampled from the Verilogue Point-of-Practice™ database of audio-recorded clinical encounters (http://www.verilogue.com). Details about the sample have been published elsewhere [18]. Briefly, physicians were recruited randomly by Verilogue from available lists and paid to record routine clinic visits with their patients. Physicians recruited patients sequentially and obtained consent prior to the recorded visit using a double opt-in method. Patients were not compensated for their participation. Physicians and patients were unaware of specific research questions for which their recorded visits would be used.

We obtained from Verilogue full visit transcripts from the most recent 1,000 encounters for management of breast cancer, major depressive disorder, and rheumatoid arthritis. These particular disease states were chosen because they are prevalent, can be managed with diagnostic and therapeutic options varying widely in cost, and feature the different clinical contexts of a potentially life-threatening disease, a mental illness, and a chronic, debilitating disease. We excluded visits with non-physician providers, those occurring outside of the United States, and those that included only physician dictations or discussions of axial spondyloarthropathy (instead of rheumatoid arthritis).

All protected health information was removed during the transcription process. The Duke University Institutional Review Board approved this study.

### Surveying extant cost conversation definitions

As a preliminary step in cost conversation definition development, we performed a literature review to identify existing definitions and criteria. We searched for English language articles published in PubMed, EMbase, Cochrane Database, and the Social Science Research Network using terms synonymous with ‘out-of-pocket costs’ or ‘cost discussion’. We reviewed titles and abstracts from resultant articles, retaining those that addressed physician-patient communication about healthcare costs. We further investigated all pertinent references in those articles to find additional studies not included in the above databases. In line with our overarching research aims, we focused our analysis on investigations reporting estimates of cost conversation incidence in U.S.-based study populations. We identified 19 studies meeting these criteria, and have described their study populations, clinical settings, cost conversation definitions, and incidence estimates in Table 1. Notably, there have been additional studies of physician-patient communication that did not report incidence estimates, but surveyed patient and/or physician attitudes about cost conversation [19–22], recommended communication strategies for cost conversation [8, 23, 24], and provided clinical and ethical perspectives on the topic [10, 11, 25]. Although these articles provided helpful context for the present investigation, they did not feature cost conversation definitions different from those detailed in Table 1. Further discussion of their content is beyond the scope of the present review.

**Table 1:** Published estimates of physician-patient cost conversation incidence with accompanying definitions

First author, year, (N)	Clinical setting and population characteristics	Cost conversation definition or criteria	Estimated cost conversation incidence
Analyses of recorded dialogue from clinic visits			
Hunter, 2016 [18] *N* = 1,755	Outpatient visits taking place in community-based practices nationwide from 2010–2014 for routine management of breast cancer, rheumatoid arthritis, and major depressive disorder. Encounters featured specialist physicians and patients >18 years old.	• Any mention of the patient’s financial costs or insurance coverage related to a specific intervention.	Overall, 30 % of clinic visits contained cost conversation. Cost conversations were observed in 22 % of breast oncology visits, 33 % of rheumatoid arthritis visits, and 38 % of depression visits.
Tarn, 2013 [38] *N* = 1,477	Outpatient visits with primary care providers occurring in a variety of locations around the U.S. from 1998–2010.	• Discussion of themes concerning cost and/or affordability discussed.	Cost and/or affordability was mentioned for 4.2 % of dietary supplements discussed.
Beard, 2010 [33] *N* = 200	Rheumatoid arthritis patients visiting rheumatologists from 2003–2005 as part of randomized controlled trial to improve communication about quality of life and medication concerns in rheumatology visits.	• Discussion of medication-related costs; could be ‘explicit’ (e.g. price of intervention) or ‘implicit’ (e.g. copay assistance, insurance coverage).	34 % of visits included discussion of medication-related costs.
Tarn, 2006 [32] *N* = 185	Outpatient visits to family physicians, internists, and cardiologists as part of the Physician Patient Communication Project (1999).	• Discussion of themes concerning medication cost and insurance.	15 % of visits with newly prescribed medications contained discussion of cost or insurance coverage.
Survey studies in non-subspecialty settings			
Alexander, 2003 [13] *N* = 484	General internists and their patients residing in the Greater Chicago area.	• Discussion of out-of-pocket (OoP) costs.	15 % of patients discussed OoP costs with their physicians.
			35 % of physicians discussed OoP costs with these patients.
Piette, 2004 [16] *N* = 660	Elderly adults with chronic illnesses who reported underusing medication in the prior year because of cost.	• Any conversation about problems paying for prescription medications.	65 % of patients with cost-related medication underuse discussed the cost problem with their physician in the prior year.
Shrank, 2006 [30] *N* = 509	Random sample of physician members of the California Medical Association. Participating physicians practiced in a variety of primary care and specialty settings.	• Discussion of OoP costs (defined as what the patient pays) or total costs with patients.	15 % of physicians reported discussing OoP costs most or all the time; 5 % reported total costs most or all the time. 45 % reported discussing OoP cost seldom or never.
Shrank, 2006 [44] *N* = 1707	Nationwide sample of patients managed by family medicine or general practitioners.	• Discussion of OoP costs.	33 % of patients with incentive-based pharmacy benefit designs discussed OoP costs with their physicians.
Wilson, 2007 [39] *N* = 17,569	Nationwide sample of Medicare beneficiaries aged 65 years or older.	• Talking with any doctor about the cost of prescription medicines in the previous 12 months.	Overall, 31 % of seniors talked with at least one of their physicians about medication costs. 61 % of those reporting cost-related non-adherence discussed cost with at least one of their physicians.
Beran, 2007 [31] *N* = 678	Internal medicine and family physicians caring for senior patients, practicing clinical medicine, and residing in California.	• Discussion of OoP cost of medications.	43 % of physicians reported discussing medication costs with at least half of their senior patients in the previous 30 days.
Tseng, 2007 [47] *N* = 1,116	Seniors enrolled in a Medicare managed care plan, surveyed in 2002. Half exceeded caps on their drug benefits the previous year, and all had total drug expenditures in the top quartile of members in their cap level.	• ‘Has your provider ever asked you whether you can afford the cost of your medications?	Overall, 17 % said providers asked about affordability; 19 % said providers usually or always discussed prices when he or she writes a prescription.
		• “How often do you and your provider talk about the price of a medication when he or she writes you a prescription?”	
Tseng, 2010 [28] *N* = 5,085	Patients with diabetes mellitus enrolled in multi-center study of diabetes care in managed care settings.	• Discussion of medication costs.	19 % of patients who reported being open to discussing cost trade-offs (discussing lower cost drugs with higher chance of adverse effects, lower effectiveness, or higher dosing frequency) said their physician usually or always discussed drug costs when prescribing.
Schmittdiel, 2010 [15] *N* = 1,458	Patients with diabetes mellitus enrolled in multi-center study of diabetes care in managed care settings.	• Talking about the amount paid for prescription drugs during 2006.	44 % of patients discussed prescription drug costs with any doctor during 2006.
Survey studies in subspecialty settings			
Schrag, 2007 [27] *N* = 167	Random, nationwide sample of medical oncologists practicing in the U.S.	• Discussion of the costs of cancer treatment.	42 % of oncologists reported discussing cost always or most of the time, 32 % reported sometimes discussing cost with their patients.
Patel, 2009 [45] *N* = 343	Random, nationwide sample of pediatricians and family physicians who care for children with asthma in the U.S.	• Asking the child’s family about OoP costs a newly prescribed asthma medication.	50 % of physicians reported asking about cost regularly when prescribing new asthma medications for children.
Neumann, 2010 [26] *N* = 787	Random sample of U.S. based oncologists with an oversample from California.	• Discussion of cancer treatment costs.	43 % always or frequently discussed cancer treatment costs with their patients, while 37 % said they did so occasionally.
Irwin, 2014 [14] *N* = 134	Breast cancer patients at a single academic cancer center.	• Discussion of costs of care.	14 % discussed costs with their doctor.
Patel, 2014 [46] *N* = 422	African-American adult women with persistent asthma recruited from a single academic center.	• Did you talk with your doctor about out-of-pocket healthcare costs?	39 % have discussed costs with their doctors.
Bestvina, 2014 [29] *N* = 300	Cancer patients in quaternary referral cancer center and affiliated rural oncology practices.	• Discussion of OoP costs of cancer care with oncologist.	19 % discussed the cost of cancer care with their oncologists.

We searched PubMed, EMbase, Cochrane Database, and the Social Science Research Network using terms synonymous with ‘*out-of-pocket costs’* and ‘*cost discussion’*. We reviewed titles and abstracts from resultant articles, retaining those that reported on the frequency of physician-patient communication about healthcare costs. We further investigated all pertinent references in these articles to identify additional studies not included in the above databases. This analysis focused on cost conversation incidence in the United States; thus, studies with international sample populations were excluded. *Abbreviations*: *OoP* indicates out-of-pocket

As seen in Table 1, substantial heterogeneity in cost conversation definitions were observed throughout studies reporting incidence estimates. For example, some studies asked participants whether they discussed cost of medications or treatment, but gave no definition or criteria to help inform them about what it meant to discuss cost [14, 26–28]. Other studies specifically asked about ‘out-of-pocket’ costs, but did not define the concept further [13, 29-31]. Some studies queried about discussion of patient costs [15, 16], while others did not specify whose cost was of interest [14]. Four prior studies performed mixed-methods analysis of cost conversation dialogue and provided examples of cost conversations; [18, 32, 33, 38] however, their analytic foci were not the cost conversation definitions themselves, and thus omitted detailed discussion of their genesis and nuance. Accordingly, we sought to develop novel, rigorous cost conversation definitions for our own analyses [18].

### Qualitative content analysis

Though review of the cost conversation literature failed to produce a guiding theoretical framework for our analysis, examples from prior work in combination with our collective experience in healthcare and healthcare analytics provided a starting point for our study. Keywords such as ‘cost’, ‘money’, ‘expense’, ‘insurance’, etc. served as indicators of a potential cost discussion, but could not be used to exclusively or conclusively identify a cost conversation. For these reasons, we approached the qualitative portion of our mixed-method study using an inductive application of summative content analysis [34]. Our method was summative in that it relied on a base understanding of how cost might be discussed and proceeded with the intent to quantify observations of such instances. However, it was still inductive in that our cost definitions were created through iterative passes through the data described in phases 1–3 below.

To mitigate bias and maximize the breadth and depth of cost conversation analysis, a multi-disciplinary group was involved in this process: physicians with extensive clinical and research experience (S.Y.Z., P.A.U.), medical students (W.G.H., A.H., C.Z.Z.), trained conversation analysts (J.K.D, L.D.W., A.H.), undergraduate students (A.W., O.C.), and graduate students training in public policy (C.C., R.F.) and marketing (D.B.). By incorporating individuals with diverse backgrounds in all phases of the analysis, we enhanced both the recognition and interpretation of cost conversations, creating an environment in which clinicians, social scientists, and policy specialists contributed equally in the multiple rounds of data-driven discussion.

#### Phase 1: immersion in the data

The foundational first step in cost conversation definition development involved immersion in the data [35]. Through close reading, we obtained a general understanding of cost conversations in their entirety, and allowed relevant themes and phenomena to emerge from the text. Themes, phenomena, and general observations were discussed in group settings and collected for subsequent definition development. Approximately 100 transcripts containing one or more keywords related to finances (e.g. expensive, cheap, insurance, dollars, co-pay, Medicare, deductible, costly, etc.) were used in this phase.

#### Phase 2: definition development

**Fig. 1:**
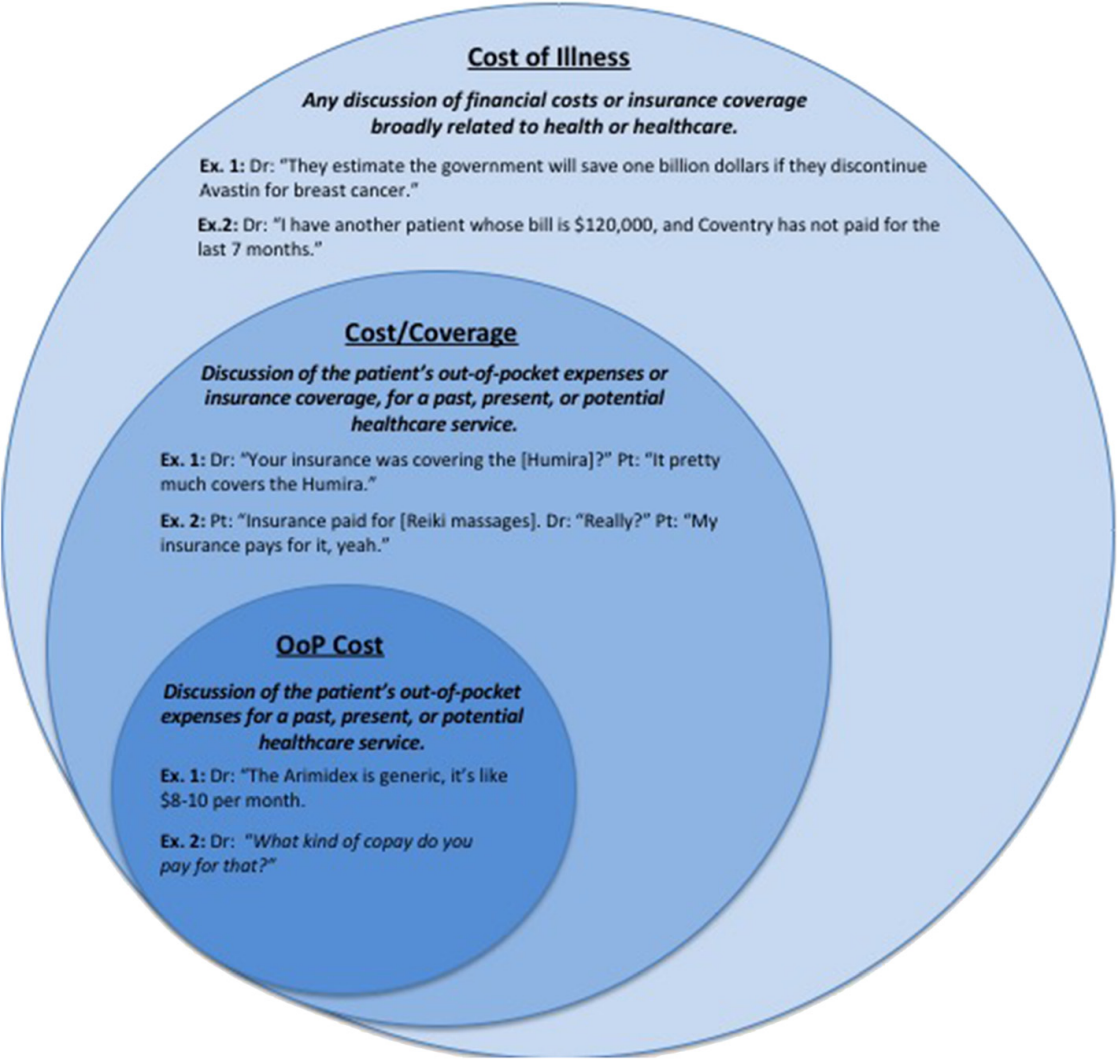
Three cost conversation definitions with explanations and example quotes

Based on phase 1 observations, clinical experience, and research experience, an initial set of definitions was created to classify relevant cost conversation types. Using analytic induction, we adjusted, merged, and split definitions until they represented distinct cost conversation types. Final definitions with accompanying examples are described in results text and shown in Fig. 1.

#### Phase 3: definition implementation

To reduce individual coder bias and improve accuracy, encounters were analyzed independently by at least two team members of our multidisciplinary team (J.K.D., C.K, L.D.W., C.Z.Z., W.G.H., P.A.U., A.W., C.C., R.F., T.T.) to determine presence or absence of cost conversations using each of the final definitions. All decisions were assessed for agreement; in cases of agreement, the corresponding decision was assigned as ‘final’. When discrepant, the final decision was made by group consensus. Team members with clinical experience (P.A.U., W.G.H.) supervised discrepancy resolution to ensure proper interpretation of clinical matters. All coding was applied using NVivo software (QSR International Pty Ltd. Version 10, 2014).

### Statistical analysis

With one recording per patient, the unit of measurement was the singular visit. Ninety-five percent confidence intervals for all proportions were calculated using Clopper and Pearson’s exact method [36]. Pearson’s Chi-Square test was used to compare cost conversation incidence estimates. R software (R Core Team (2013), Version 3.0.1, http://www.R-project.org/) was used to perform all statistical analyses. All authors had full access to the data, and take responsibility for its integrity.

## Results

### Study population

Patient, physician, and visit characteristics are presented in Table 2 and described in detail elsewhere [18]. A total of 118 unique physicians comprised of *N* = 56 oncologists, *N* = 36 psychiatrists, and *N* = 26 rheumatologists recorded visits for this study. The median number of visits recorded by each physician was 12 (interquartile range (IQR), 4 to 22). Notable differences between clinical settings included a higher percentage of male physicians in major depression visits (100 %), higher median number of visits recorded by each rheumatologist (28; IQR 5–41), and higher percentage of female patients in the breast oncology setting (99 %). For detailed information about clinic visit locations, see Additional file 1: Table S1.

**Table 2:** Physician, patient, and visit characteristics^a^

Characteristics	Breast cancer	Depression	Rheumatoid arthritis
Patients (N)	677	422	656
Age, years (%)			
19–34	1	23	5
35–54	28	44	29
55–74	54	33	53
75+	17	1	13
Gender, female (%)	99	66	78
Race (%)			
Caucasian	71	83	75
African– American	19	7	11
Hispanic	6	5	9
Other	4	4	5
Insurance status (%)			
Private	49	49	54
Public	51	43	41
Uninsured	0	8	4
Physicians (N)	56	36	26
Visits recorded, median (N), (IQR)	9 (5–19)	13 (4–17)	28 (5–41)
Gender, male (%)	89	100	81
Years in practice (%)			
0–10	37	16	11
11–20	44	34	58
21–30	15	44	14
31+	4	6	16
Visit dates	Jun 2010 – Aug 2013	May 2010– Jan 2014	Mar 2012 – Feb 2014
Visit Locations by US region^b^ (N)			
East North Central	98	105	47
East South Central	23	21	69
Middle Atlantic	56	85	100
Mountain	80	22	0
New England	41	39	20
Pacific	74	18	177
South Atlantic	222	41	50
West North Central	23	55	102
West South Central	60	36	91

^a^Values are percentages of total non–missing observations unless otherwise indicated. Less than 1 % of observations are missing. Percentage totals do not all sum to 100 due to rounding

^b^List of states comprising each United States geographic region is provided in the Additional file 1: Table S1. *Abbreviation*: *IQR* indicates inter–quartile range

### Three definitions of ‘cost conversation’

Three definitions of cost conversation emerged from our analysis. These definitions were hierarchical; all dialogue considered cost conversation using the narrowest definition (addressing the patient’s out-of-pocket healthcare costs; OoP cost), was also considered cost conversation using the broader definition (addressing patient’s out-of-pocket costs or insurance coverage; Cost/Coverage). Dialogue counted as cost conversation by the OoP Cost or Cost/Coverage definitions was also considered cost conversation using the broadest definition (conversations about financial costs or insurance coverage broadly related to health or healthcare; Cost of Illness). These definitions are described in more detail below with illustrative examples in the text and in Fig. 1.

#### OoP cost: conversation about the patient’s out-of-pocket costs

The OoP Cost definition constrained the concept of cost conversation to discussions about the patient’s out-of-pocket costs for past, present, or potential healthcare services. In alignment with the Agency for Healthcare Research and Quality (AHRQ), out-of-pocket costs were defined as the portion of total healthcare expenses paid by the patient (not including payments made for health insurance premiums) [37]. In contrast to some prior studies, we had no a priori emphasis on cost conversations related to particular types of interventions, (e.g. prescription medications, dietary supplements, domain-specific treatments such as chemotherapy related costs); [15, 29, 38] thus, we included conversations addressing the patient’s out-of-pocket costs for any healthcare service. Specifically, healthcare services were defined as any diagnostic or therapeutic modality intended to assess or alter a patient’s disease course or health-related quality of life. Accordingly, we included discussions of patient out-of-pocket costs for a variety of interventions, including, but not limited to: pharmacotherapies (e.g. prescription medications, over-the-counter medications such as non-steroidal anti-inflammatory agents, fish oil, folic acid, vitamin D); diagnostic tests (e.g. CT scan, complete blood count, OncoType DX, BRCA testing); services from non-physician healthcare providers (e.g. dental care, wound management, cognitive behavioral therapy, podiatric care); and additional therapies such as acupuncture, nutritional supplements, or massage, when provided for medical but not recreational purposes (e.g. acupuncture for treatment of chronic pain, nutritional supplement for cachexia or malnutrition).

We observed a few common forms of dialogue meeting the OoP Cost definition. The clearest form of OoP Cost conversation was when the patient’s out-of-pocket costs were explicitly discussed (e.g. Dr: “*How much copay do you have for that?*”), or described with an exact dollar value (e.g. Dr: “*With Methotrexate, you’re looking at about $20 per month*”). Another common form of OoP Cost conversation occurred when one of the parties described the patient’s out-of-pocket costs with a subjective description (e.g. Dr: *“Do you want to take biologics?* Pt: “*I don't have very good insurance. I have to pay 50 % and it costs a lot.*”).

In some cases, costs were discussed without any explicit textual evidence indicating whether they were specifically the patient’s out-of-pocket costs, as opposed to the costs born by other stakeholders, such as insurance companies or the healthcare system as a whole. Rather than exclude all such dialogue without explicit literal cues (e.g. Pt: “*Can we do the 3 month supply of that? It’s cheaper that way.*”), we carefully evaluated the context of the dialogue to determine its implicit meaning. For example, in the latter quote, the patient does not explicitly state for whom the 3-month supply will be cheaper; however, she made no other comments about insurance companies or the healthcare system as a whole throughout the visit. In the absence of any evidence suggesting that the patient was considering other stakeholders’ costs, we inferred that she was discussing her own out-of-pocket costs (i.e. it’s cheaper that way [for me]).

Another common form of dialogue meeting the OoP Cost definition was discussion of strategies by which the patient’s out-of-pocket costs could be reduced. These discussions did not always contain explicit textual references to the patient’s out-of-pocket costs (e.g. Dr: “*We’ll get you set up with the copay assistance program for Xeloda before you leave today.*”); however, they were still considered OoP Cost conversations based on the notion that discussion of cost-reducing strategies for the patient constitute indirect, implied discussion of their out-of-pocket costs. For example, in the latter quote, by offering to ‘set [the patient] up with the copay assistance program’, the physician was implicitly stating that he would attempt to lower the patient’s out-of-pocket costs for Xeloda. See Fig. 1 for more examples.

#### Cost/coverage: conversation addressing patients’ out-of-pocket costs OR insurance coverage

As depicted in Fig. 1, dialogue considered cost conversation using the OoP Cost definition was also considered cost conversation using the Cost/Coverage definition. The Cost/Coverage definition broadens the concept of ‘cost conversation’ beyond the OoP Cost definition by also including discussions about insurance coverage (e.g. Pt: “*Do you think that we’ll be able to get the Enbrel covered?”*). We observed a few common forms of dialogue addressing insurance coverage throughout our sample: 1) discussion of presence or absence of insurance coverage for interventions previously ordered (e.g. Dr: “*Has your insurance been covering the Abilify*?”) or interventions under consideration which the patient had never been prescribed (e.g. Dr: “*I’m going to talk to [my staff] and see if we can actually get this [Faslodex] approved by your insurance, today or tonight.*”); and 2) discussion about the perceived quality or degree of coverage (e.g. Dr: *“So you have pretty good insurance through Piedmont?”*).

Importantly, discussions about insurance coverage included in the Cost/Coverage definition were required to be related to a *particular* healthcare service received by the patient (Dr: “*We can do the paperwork so that the insurance company approves [the lab studies] before we do them… You have insurance, right*?” Pt: “*Yeah*.”). By contrast, discussion of insurance coverage that was not tied to a specific intervention (e.g. physician queries about patients’ insurance status occurring at the beginning of clinic visits as part of routine demographic data collection) was not included as cost conversation using the Cost/Coverage definition.

#### Cost of illness: conversation about financial costs or insurance coverage broadly related to health or healthcare

Dialogue considered cost conversation using the OoP Cost or Cost/Coverage definitions was also included as cost conversation using the Cost of Illness definition. Additionally, though, the Cost of Illness definition broadened the concept of cost conversation in a few important ways. First, the Cost of Illness definition included discussions of healthcare costs born by any stakeholder (not just the patient). Thus, discussions of healthcare costs born by family members, friends, or society as a whole were considered cost conversations using this definition (e.g. Dr: *The FDA pulled Avastin because it's an expensive drug, a very expensive drug. Thousands and thousands per month, and if no one’s living longer, what’s the point of our health care system spending all the money on it*?”).

Second, the Cost of Illness definition broadened the concept of cost conversation by including discussions of insurance coverage that were not related to a specific intervention, as in the aforementioned physician queries about insurance status occurring at the beginning of some clinic visits (e.g. Dr: *“You live with your daughter? And you are Hispanic? You’re on Medicaid?”*). These queries were often brief, and resultant information was not pursued further to assess or manage the patient’s healthcare costs. This inclusion reflects and implements the perspective that any discussion of insurance coverage, no matter the context, addresses the patient’s costs.

Lastly, the Cost of Illness definition broadened the concept of cost conversation by also including those discussions in which the physician and patient addressed secondary financial consequences of missing work due to treatment side effects. For example, some breast cancer patients complained their chemotherapy was causing them either to lose wages or become at risk for termination and loss of health insurance coverage. Since these situations did not address the costs the patient would pay for a health care intervention, they did not meet the criteria for the OOP Cost or Cost/Coverage definitions; however, they did address financial costs related to the patient’s health problems, and were thus included in the Cost of Illness definition.

#### Excluded from all definitions

We excluded several types of conversation from all three definitions: discussions of non-healthcare related costs (e.g. children’s college tuition), general financial issues not explicitly mentioned to be impacting the patient’s ability to access or afford medical care (e.g. receiving a pay cut), or the logistics of obtaining an intervention (e.g. recommending a specific pharmacy to fill without mentioning any corresponding cost savings).

### Incidence of cost conversations

#### Published estimates of cost conversation incidence

Our review demonstrated that estimates of cost conversation incidence vary widely in the published literature (Table 1). Incidence estimates range from 14–19 % of oncology patients *ever* discussing healthcare costs with physicians [14, 29], to 44 % of diabetic patients discussing prescription drug costs with their physicians in the previous year [15], to 65 % of patients with cost-related non-adherence discussing healthcare costs with physicians in the previous year [16]. Despite marked heterogeneity with respect to study methodology, patient characteristics, and time frame of data collection, a few notable patterns emerged.

One such pattern is significant variation in cost conversation incidence across clinical settings. This was demonstrated in a prior investigation by our group, in which a statistically significant difference in cost conversation incidence was observed across three clinical settings, breast oncology (estimated cost conversation incidence, 22 % of clinic visits), rheumatoid arthritis (33 % of visits), and depression (38 % of visits; analysis of variance *P* < 0.001 for comparison among the three settings) [18]. Interestingly, our estimate of cost conversation incidence in the setting of rheumatoid arthritis was nearly equal to the estimate reported by Beard et al. (33 % vs. 34 % of visits, respectively), which was also produced by analysis of recorded dialogue [33]. These estimates from rheumatoid arthritis settings were generally greater than estimates from oncology settings, in which fewer than 20 % of patients reported discussing cost with their oncologists [14, 29].

Another pattern emerging in the published literature was that cost conversation incidence estimates based on physician surveys were generally higher than those based on patient surveys; this was best demonstrated in a study performed by Alexander and colleagues, in which 15 % of patients reported ever discussing cost with their providers, but 35 % of providers reported discussing cost with those same patients [13]. In other surveys, over 40 % of physicians reported that that they always or frequently discussed cost with their patients, and an additional 30 % reported having these discussions sometimes or occasionally [26, 27]. This contrasts with multiple patient surveys which reported fewer than 20 % of patients discussing cost with their physicians [14, 28, 29].

#### Cost conversation incidence in 1,755 clinic visits using three definitions

**Fig. 2:**
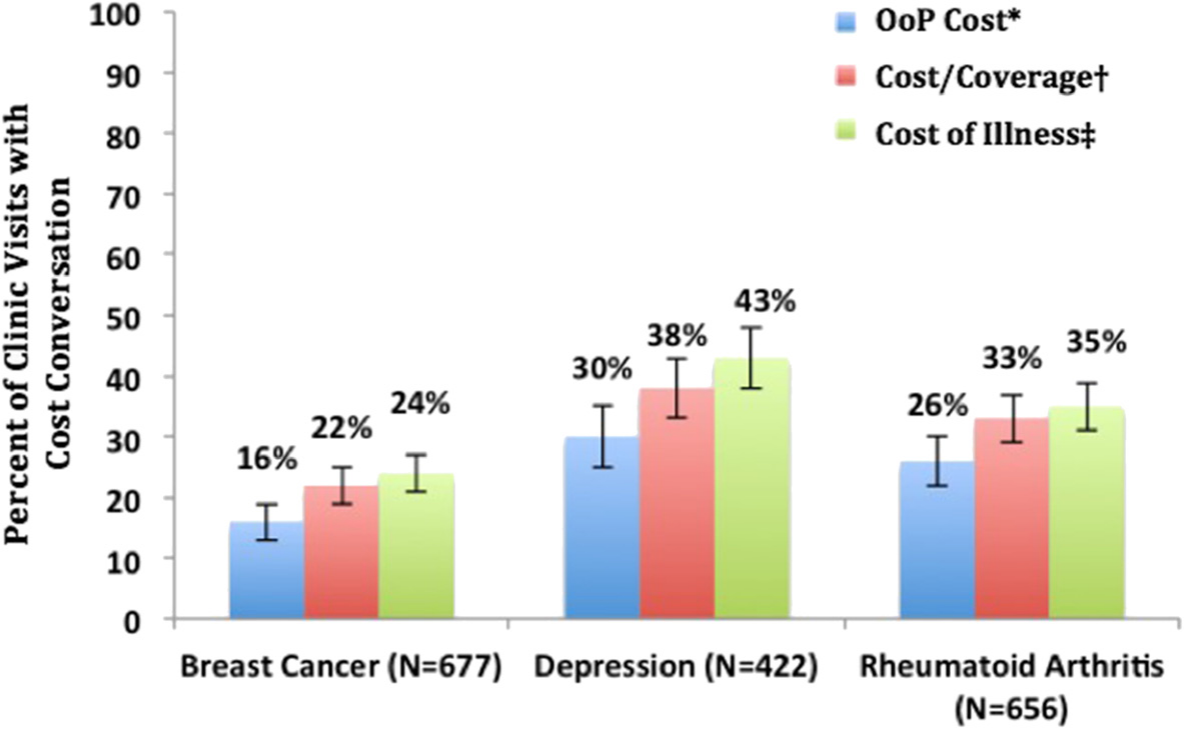
Estimated incidence of cost conversations using three different definitions. All visits featured adult patients (>18 years old) and specialist physicians. *OoP Cost: discussion of the patient’s out-of-pocket costs for healthcare services; †Cost/Coverage: discussion of the patient’s out-of-pocket costs OR insurance coverage; ‡Cost of Illness: any discussion of financial costs or insurance coverage broadly related to health or healthcare [13–16, 18, 26–33, 38, 39, 44–46]

When using the OoP Cost definition, 23 % of all visits in our sample (95 % confidence interval [CI], 21 to 25) contained a cost conversation. In the individual disease settings, cost conversations were identified in 16 % of breast oncology visits (95 % CI, 13 to 19), 30 % of depression visits (95 % CI, 25 to 34), and 26 % of rheumatology visits (95 % CI, 22 to 29; *p* < 0.001 for comparison across the three settings; Fig. 2). More specifically, dollar values were mentioned for the patient’s past, present, or potential healthcare services (e.g. Dr: “*The copay cost for Humira was $400*) in 5 % of breast cancer visits, and 10 % of rheumatoid arthritis and depression visits (*P* < 0.01 for comparison across the three clinical settings).

When using the Cost/Coverage definition, 30 % of all clinic visits (95 % CI, 28 to 32) contained a cost conversation – 22 % of breast cancer visits (95 % CI, 19 to 25), 38 % of depression visits (95 % CI, 33 to 43), and 33 % of rheumatoid arthritis visits (95 % CI, 30 to 37; *p* < 0.001 for comparison across the three settings).

When using the Cost of Illness definition, 32 % of all clinic visits (95 % CI, 30 to 34) contained a cost discussion – 24 % of breast oncology visits (95 % CI, 21 to 27), 43 % of depression visits (95 % CI, 38 to 48), and 35 % of rheumatology visits (95 % CI, 31 to 39, *p* < 0.001 for overall comparison among the three settings).

## Discussion

Estimates of physician-patient cost conversation incidence vary substantially in the published literature [13, 15, 29, 39]. Differences in study design, clinical context, and mode of inquiry (i.e. physician survey, patient survey, analysis of recorded dialogue) contribute to this heterogeneity. In this study, we found that variation in ‘cost conversation’ definition also impacts the estimated incidence of physician-patient cost conversations. Specifically, we observed significant differences between cost conversation incidence estimates when using a definition limited to explicit discussions of the patient’s out-of-pocket costs for healthcare services (OoP Cost), versus a definition that included discussions of out-of-pocket costs or insurance coverage (Cost/Coverage), versus a definition capturing all discussions broadly related to the cost of health or healthcare (Cost of Illness). Importantly, the significant impact of definition on incidence was observed in three different clinical settings, using a mode of inquiry (analysis of naturally occurring clinical dialogue), which allowed for rigorous observation of the behavior in question.

Using the OoP Cost definition, the lowest rate of cost conversation occurred in oncology clinics, where cost conversations occurred in about 1 in 6 breast cancer visits. Despite employing the strictest of the three definitions, this estimate was still higher than some previous survey estimates, which reported less than 1 in 6 breast cancer patients *ever* discussed healthcare costs with their physicians [14]. By contrast, the highest rate of cost conversation in our sample was in psychiatry clinics, where nearly half of depression visits involved cost conversations when using the Cost of Illness definition. This is noteworthy because even when broadening the definition of cost conversation to its most liberal interpretation, healthcare costs were not discussed in the majority of clinic visits. Thus, our results may suggest that physician-patient discussions about healthcare costs in outpatient settings are not as rare as some prior surveys suggested [13, 29].

These findings have important clinical and policy implications. Approximately one third of Americans are burdened by the costs of their medical care, either paying their medical bills late or not paying them at all [1, 2]. Physician-patient cost conversations have been shown to significantly increase the odds of patients receiving financial assistance, through avenues such as copay assistance programs or switching to lower cost alternative therapies [39, 40]. Additionally, 72 % of patients have reported that discussing healthcare costs with their physicians was helpful [16]. By supporting the notion that physician-patient cost conversations are not altogether rare in outpatient clinic visits, our findings may suggest that such visits are promising sites for out-of-pocket cost management. Indeed, in other analyses from these data, 44 % of physician-patient cost conversations included discussion of cost saving strategies, further highlighting the ongoing effort and potential for physician-patient cost communication in the management of patients’ healthcare related financial burden [18].

Additionally, this study has implications for research in the arena of physician-patient communication about healthcare costs. We show that the incidence of cost conversations varies significantly, depending on how researchers define “cost conversation.” This finding facilitates more meaningful synthesis and understanding of the wide range of results from prior studies (Table 1). This study also provides direction for future investigations aiming to quantify the incidence, content, or impact of cost conversations, as we provide a set of definitions that can be transferred and utilized in future studies.

Notably, each of our definitions represents a potentially valid conceptual construct of cost conversation, and is suited to particular analytic aims. For example, when evaluating the impact of cost conversations on patient adherence, financial status, or medical outcomes, investigators may wish to focus on cost conversations specifically about the patient’s costs, rather than costs to any stakeholder; thus, the Cost/Coverage or OOP Cost definitions would be preferred over the Cost of Illness definition. Additionally, since insurance coverage may constitute an important potential cost barrier for the patient, conversations about patient’s insurance coverage could also impact patient adherence or outcomes, thus making the Cost/Coverage definition a potentially optimal choice for this particular research aim.

By contrast, when investigating the sociolinguistic aspects of cost communication, investigators may wish to examine any conversation broadly related to cost. Since discussion of costs borne by family or friends and rote checks of insurance coverage are still, fundamentally, communication about healthcare costs, the Cost of Illness definition may be best suited to these types of investigations. Lastly, if investigators seek to evaluate physicians’ assessment of patients’ costs or the impact of perceived high costs on treatment decisions, they may wish to narrow their scope of cost conversation analysis to those which include some description of the magnitude of the cost. In this case, the OoP Cost definition may be best suited for the analysis in question.

One limitation of this study is the population sampled. We focused on only three disease states, chosen because they expose patients to high out-of-pocket costs [7, 29, 41–43]. These diseases represent varied contexts of a life-threatening or terminal illness (breast cancer), a chronic debilitating condition (rheumatoid arthritis), and a mental health condition (depression), thereby offering greater clinical diversity than many prior investigations on this topic. But we cannot generalize our findings to other diseases. Additionally, we did not measure cost conversation incidence for all potential definitions of ‘cost conversation; instead, we used a data-driven mixed-methods analysis to create definitions that represented distinct and clinically relevant cost conversation types. Thus, although not an exhaustive inquiry of all potential definitions, our study provides a helpful understanding of the impact of key ‘cost conversation’ definitions and concepts on estimates of cost conversation incidence. Further investigation will be needed to elucidate the complexity and nuance of physician-patient communication about healthcare costs.

## Conclusions

The frequency of physician-patient healthcare cost conversations has varied widely across studies [13, 15, 29, 39]. These reports have varied in part because the mode of inquiry has varied, with lower rates reported from surveys asking patients to recall frequency of cost discussion [13, 14, 29], and higher rates reported from analyses of actual clinical dialogue [18, 32, 33]. In this study, we demonstrate how much estimates can vary within the *same* mode of inquiry. Importantly, our mode of analysis was naturally occurring clinical dialogue, which allowed for rigorous observation of the behavior in question. Herein, we discovered that estimates of cost conversation vary significantly based on which of several possible definitions of cost conversation researchers adopt. This provides novel insights for studies on this important aspect of patient experience.

## Additional file

Additional file 1: Table S1. Location of Clinic Visits by Region and State. (DOCX 31 kb)
