# Supplementary material for: Patient-physician discussions about costs: definitions and impact on cost conversation incidence estimates
Source: BMC Health Serv Res. 2016 Mar 31;16:108. doi: 10.1186/s12913-016-1353-2 (PMC4815215; doi:10.1186/s12913-016-1353-2)
Supplement: Additional file 1: Table S1. — Location of Clinic Visits by Region and State. (DOCX 31 kb) [file 12913_2016_1353_MOESM1_ESM.docx]

Additional file 1: Table S1 **Location of Clinic Visits by Region and State**

|  | | | | |
| --- | --- | --- | --- | --- |
|  | **Breast Cancer** | **Depression** | **Rheumatoid Arthritis** | **Overall** |
| **East North Central** | **98** | **105** | **47** | **250** |
| Illinois | 13 | 45 | 0 | 58 |
| Indiana | 34 | 13 | 0 | 47 |
| Michigan | 18 | 17 | 0 | 35 |
| Ohio | 33 | 28 | 47 | 108 |
| Wisconsin | 0 | 2 | 0 | 2 |
| **East South Central** | **23** | **21** | **69** | **113** |
| Kentucky | 23 | 4 | 0 | 27 |
| Mississippi | 0 | 17 | 0 | 17 |
| Tennessee | 0 | 0 | 69 | 69 |
| **Middle Atlantic** | **56** | **85** | **100** | **241** |
| New Jersey | 29 | 19 | 0 | 48 |
| New York | 27 | 35 | 100 | 162 |
| Pennsylvania | 0 | 31 | 0 | 31 |
| **Mountain** | **80** | **22** | **0** | **102** |
| Arizona | 36 | 22 | 0 | 58 |
| Colorado | 44 | 0 | 0 | 44 |
| **New England** | **41** | **39** | **20** | **100** |
| Connecticut | 41 | 0 | 0 | 41 |
| Massachusetts | 0 | 39 | 20 | 59 |
| **Pacific** | **74** | **18** | **177** | **269** |
| California | 74 | 0 | 177 | 251 |
| Hawaii | 0 | 18 | 0 | 18 |
| **South Atlantic** | **222** | **41** | **50** | **313** |
| Delaware | 8 | 0 | 0 | 8 |
| District of Columbia | 0 | 0 | 6 | 6 |
| Florida | 68 | 0 | 33 | 101 |
| Georgia | 126 | 27 | 0 | 153 |
| Maryland | 15 | 0 | 9 | 24 |
| North Carolina | 0 | 5 | 2 | 7 |
| South Carolina | 0 | 8 | 0 | 8 |
| Virginia | 0 | 1 | 0 | 1 |
| West Virginia | 5 | 0 | 0 | 5 |
| **West North Central** | **23** | **55** | **102** | **180** |
| Iowa | 0 | 0 | 50 | 50 |
| Kansas | 0 | 22 | 0 | 22 |
| Minnesota | 0 | 33 | 0 | 33 |
| Missouri | 0 | 0 | 52 | 52 |
| South Dakota | 23 | 0 | 0 | 23 |
| **West South Central** | **60** | **36** | **91** | **187** |
| Louisiana | 0 | 0 | 42 | 42 |
| Oklahoma | 25 | 1 | 0 | 26 |
| Texas | 35 | 35 | 49 | 119 |
| **Overall** | **677** | **422** | **656** | **1755** |
